# Supplementary material for: A Likelihood Approach for Real-Time Calibration of Stochastic Compartmental Epidemic Models
Source: PLoS Comput Biol. 2017 Jan 17;13(1):e1005257. doi: 10.1371/journal.pcbi.1005257 (PMC5240920; doi:10.1371/journal.pcbi.1005257)
Supplement: S1 File — (TAR.GZ) [file pcbi.1005257.s014.tar.gz › HSPH_Online-SI-Revision/MSS10/n100-extreme/n100-extreme_NEW2_table-8.pdf]

1	newly 3 weeks spec	1767.	2792.38	{2160., 3360.}	59.9593	0.20018	newly 3 weeks cumul	3085.	4540.51	{3556., 5443.}	49.1335	0.170362	newly 5 weeks spec	5804.	9636.28	{7677., 11 081.}	67.378	0.220511	newly 5 weeks cumul	12 241.	19 853.2	{15 741., 23 246.}	63.7027	0.210847	newly 62 weeks cumul	91 729.	82 120.5	{56 741., 89 482.}	10.5406	0.0533902
2	newly 3 weeks spec	2838.	3236.6	{2198., 4429.}	26.6406	0.104979	newly 3 weeks cumul	5925.	6751.37	{4655., 9435.}	25.7669	0.101591	newly 5 weeks spec	5914.	4912.67	{3897., 5964.}	17.5663	0.088881	newly 5 weeks cumul	16 050.	15 969.5	{11 552., 20 450.}	17.228	0.0770276	newly 58 weeks cumul	82 551.	34 997.8	{33 122., 36 676.}	57.6047	0.373045
3	newly 3 weeks spec	1557.	3695.15	{2062., 5582.}	139.347	0.353913	newly 3 weeks cumul	2607.	5444.09	{3135., 7994.}	110.842	0.302294	newly 5 weeks spec	6571.	14 044.3	{9094., 18 542.}	116.356	0.328458	newly 5 weeks cumul	12 454.	28 015.4	{16 949., 39 107.}	127.318	0.343797	newly 63 weeks cumul	90 095.	78 941.9	{70 541., 85 636.}	12.5128	0.0609645
4	newly 3 weeks spec	302.	392.14	{13., 827.}	113.636	0.618223	newly 3 weeks cumul	421.	523.56	{35., 1101.}	103.796	0.507636	newly 5 weeks spec	3492.	3253.99	{39., 8367.}	95.7196	0.857062	newly 5 weeks cumul	5026.	5125.49	{154., 12 232.}	100.763	0.701557	newly 67 weeks cumul	90 300.	74 274.	{37 228., 92 640.}	19.1072	0.33031
5	newly 3 weeks spec	841.	293.35	{42., 569.}	66.239	0.664997	newly 3 weeks cumul	1316.	573.3	{123., 1133.}	58.4954	0.50855	newly 5 weeks spec	3644.	789.87	{49., 1613.}	79.1369	0.965205	newly 5 weeks cumul	6776.	1844.98	{227., 3576.}	73.6092	0.763461	newly 64 weeks cumul	88 461.	36 416.4	{13 465., 53 504.}	58.8723	0.530509
6	newly 3 weeks spec	1468.	1243.98	{946., 1571.}	19.3174	0.0970063	newly 3 weeks cumul	2440.	2159.28	{1722., 2645.}	16.4836	0.0799465	newly 5 weeks spec	5118.	4273.72	{3153., 5643.}	21.1055	0.109038	newly 5 weeks cumul	10 351.	8827.42	{6701., 11 397.}	19.6702	0.099352	newly 62 weeks cumul	91 404.	90 730.1	{83 163., 93 334.}	3.21683	0.0144833
7	newly 3 weeks spec	1624.	1044.66	{851., 1247.}	35.7032	0.19702	newly 3 weeks cumul	2704.	1879.06	{1559., 2209.}	30.6457	0.16329	newly 5 weeks spec	5875.	3355.44	{2746., 3893.}	42.8861	0.24837	newly 5 weeks cumul	11 979.	7157.84	{5901., 8267.}	40.2468	0.228664	newly 60 weeks cumul	90 884.	84 731.1	{77 366., 92 377.}	7.78769	0.0359203
8	newly 3 weeks spec	1359.	1776.8	{1390., 2108.}	32.2296	0.117754	newly 3 weeks cumul	2461.	2978.67	{2348., 3524.}	23.4754	0.0894252	newly 5 weeks spec	5170.	6409.02	{5035., 7682.}	26.0623	0.0979218	newly 5 weeks cumul	10 407.	12 960.3	{10 227., 15 557.}	26.4921	0.0993406	newly 62 weeks cumul	92 361.	84 862.2	{77 413., 89 856.}	8.2194	0.0382397
9	newly 3 weeks spec	953.	566.79	{408., 715.}	40.5257	0.236467	newly 3 weeks cumul	1486.	1008.1	{756., 1240.}	32.2921	0.178144	newly 5 weeks spec	4055.	2003.83	{1391., 2601.}	50.5837	0.31885	newly 5 weeks cumul	7477.	4095.22	{2842., 5185.}	45.2291	0.27296	newly 62 weeks cumul	92 411.	84 582.6	{77 539., 93 388.}	9.04667	0.0423703
10	newly 3 weeks spec	629.	1352.64	{388., 2464.}	132.967	0.371777	newly 3 weeks cumul	952.	1849.16	{586., 3298.}	112.307	0.331391	newly 5 weeks spec	4393.	9765.14	{2801., 15 536.}	139.192	0.405247	newly 5 weeks cumul	6990.	15 814.2	{4579., 26 394.}	143.041	0.399824	newly 66 weeks cumul	75 317.	85 516.4	{70 827., 93 952.}	18.0267	0.0762789
11	newly 3 weeks spec	2514.	1735.9	{1187., 2388.}	32.2299	0.184211	newly 3 weeks cumul	4845.	3588.6	{2465., 4799.}	28.5461	0.157082	newly 5 weeks spec	6044.	3315.55	{2432., 4191.}	45.1431	0.272848	newly 5 weeks cumul	15 023.	9402.6	{6688., 12 448.}	37.6252	0.218259	newly 60 weeks cumul	84 746.	37 042.1	{33 133., 40 350.}	56.2904	0.361274
12	newly 3 weeks spec	1011.	412.19	{319., 480.}	60.3393	0.406251	newly 3 weeks cumul	1634.	728.87	{574., 832.}	56.265	0.363181	newly 5 weeks spec	4508.	1506.69	{1112., 1733.}	68.7491	0.505981	newly 5 weeks cumul	8400.	3032.47	{2250., 3494.}	65.6973	0.466618	newly 61 weeks cumul	92 398.	91 710.9	{89 724., 93 648.}	2.33163	0.0103856
13	newly 3 weeks spec	2062.	1810.19	{1418., 2218.}	15.2459	0.077712	newly 3 weeks cumul	3828.	3508.11	{2897., 4247.}	13.0337	0.0643324	newly 5 weeks spec	4926.	4337.8	{3132., 5275.}	15.853	0.0821642	newly 5 weeks cumul	12 079.	10 735.3	{8187., 12 864.}	14.728	0.074946	newly 59 weeks cumul	90 371.	64 418.5	{39 128., 72 145.}	28.7177	0.15835
14	newly 3 weeks spec	207.	683.84	{0., 1122.}	339.188	1.14658	newly 3 weeks cumul	282.	915.4	{17., 1474.}	308.723	0.642969	newly 5 weeks spec	1855.	2062.5	{0., 10 499.}	156.502	1.72527	newly 5 weeks cumul	2833.	4547.52	{17., 16 550.}	194.939	1.11255	newly 64 weeks cumul	71 218.	21 856.2	{17., 49 912.}	69.3109	1.3803
15	newly 3 weeks spec	874.	505.11	{345., 707.}	42.2414	0.254055	newly 3 weeks cumul	1434.	842.01	{567., 1148.}	41.2824	0.245222	newly 5 weeks spec	4922.	2175.59	{1441., 2989.}	55.7987	0.37649	newly 5 weeks cumul	8480.	4077.17	{2703., 5524.}	51.9202	0.33669	newly 64 weeks cumul	79 509.	85 290.3	{71 366., 93 796.}	12.0233	0.0506076
16	newly 3 weeks spec	582.	76.28	{24., 135.}	86.8935	0.978617	newly 3 weeks cumul	805.	135.99	{49., 229.}	83.1068	0.847469	newly 5 weeks spec	4823.	295.87	{74., 544.}	93.8654	1.35564	newly 5 weeks cumul	7337.	581.38	{178., 1037.}	92.0761	1.21443	newly 64 weeks cumul	91 266.	84 718.5	{73 607., 93 241.}	8.2979	0.0397237
17	newly 3 weeks spec	2320.	3471.01	{3205., 3707.}	49.6125	0.174443	newly 3 weeks cumul	4411.	6284.03	{5886., 6616.}	42.4627	0.153256	newly 5 weeks spec	5419.	8563.38	{8211., 8808.}	58.0251	0.198557	newly 5 weeks cumul	13 371.	20 698.9	{19 631., 21 552.}	54.8041	0.189512	newly 59 weeks cumul	87 876.	91 940.4	{88 038., 93 129.}	4.63253	0.0195574
18	newly 3 weeks spec	1347.	1441.96	{1294., 1587.}	10.1886	0.042651	newly 3 weeks cumul	2379.	2532.3	{2329., 2763.}	9.05338	0.0379925	newly 5 weeks spec	4153.	4671.27	{4267., 5080.}	14.5122	0.0592445	newly 5 weeks cumul	9012.	9897.1	{9056., 10 829.}	12.0806	0.0499511	newly 60 weeks cumul	79 943.	92 802.3	{92 715., 93 574.}	16.0855	0.0646837
19	newly 3 weeks spec	927.	890.67	{538., 1315.}	33.6516	0.151507	newly 3 weeks cumul	1494.	1426.57	{913., 2045.}	30.2068	0.137458	newly 5 weeks spec	4254.	4155.41	{2258., 6725.}	39.09	0.17442	newly 5 weeks cumul	7918.	7610.05	{4260., 11 881.}	36.5404	0.164841	newly 62 weeks cumul	93 229.	83 217.	{70 745., 91 984.}	10.7595	0.0528999
20	newly 3 weeks spec	53.	74.06	{1., 194.}	126.943	0.70536	newly 3 weeks cumul	74.	113.35	{6., 278.}	128.5	0.526701	newly 5 weeks spec	1059.	569.14	{0., 1565.}	93.2559	1.2413	newly 5 weeks cumul	1395.	892.94	{9., 2352.}	94.6667	0.963218	newly 67 weeks cumul	91 568.	69 809.	{20., 92 875.}	24.3288	0.559006
21	newly 3 weeks spec	845.	781.07	{427., 1219.}	34.7183	0.174081	newly 3 weeks cumul	1345.	1258.86	{730., 1835.}	31.545	0.156847	newly 5 weeks spec	4282.	3607.24	{1745., 5729.}	40.0084	0.215963	newly 5 weeks cumul	7677.	6620.93	{3488., 10 252.}	38.1413	0.201639	newly 64 weeks cumul	83 732.	83 925.6	{60 232., 93 213.}	11.1902	0.0525442
22	newly 3 weeks spec	804.	704.9	{108., 979.}	85.9826	0.480179	newly 3 weeks cumul	1237.	1042.27	{228., 1492.}	75.4188	0.404384	newly 5 weeks spec	4322.	3004.93	{372., 5089.}	81.0798	0.597259	newly 5 weeks cumul	7480.	5776.89	{830., 8724.}	83.852	0.525682	newly 65 weeks cumul	84 795.	65 371.5	{36 406., 91 257.}	26.3191	0.187148
23	newly 3 weeks spec	2606.	2216.6	{1463., 2833.}	23.0115	0.116814	newly 3 weeks cumul	5006.	4407.96	{3043., 5698.}	19.8594	0.0985604	newly 5 weeks spec	5905.	4572.8	{3090., 5891.}	27.5296	0.147105	newly 5 weeks cumul	15 055.	12 336.5	{8254., 15 725.}	24.0038	0.124156	newly 58 weeks cumul	84 036.	45 267.2	{36 455., 54 735.}	46.1336	0.275646
24	newly 3 weeks spec	1803.	1436.41	{261., 2602.}	41.5025	0.324436	newly 3 weeks cumul	3172.	2520.03	{487., 4505.}	39.4398	0.282224	newly 5 weeks spec	5220.	4389.44	{688., 7457.}	41.2268	0.332683	newly 5 weeks cumul	11 528.	9573.22	{1797., 16 532.}	40.4519	0.299778	newly 58 weeks cumul	92 404.	48 677.1	{34 922., 57 196.}	47.3214	0.345158
25	newly 3 weeks spec	784.	349.12	{3., 764.}	71.0893	0.915709	newly 3 weeks cumul	1188.	621.91	{26., 1306.}	64.8577	0.627749	newly 5 weeks spec	4655.	1137.48	{0., 2640.}	83.8415	1.42473	newly 5 weeks cumul	7743.	2435.18	{36., 5536.}	78.5644	0.992819	newly 61 weeks cumul	89 551.	21 761.6	{36., 37 672.}	75.6993	1.12597
26	newly 3 weeks spec	2337.	2379.41	{1515., 2984.}	20.5738	0.0925918	newly 3 weeks cumul	4561.	4628.21	{3027., 5709.}	17.6661	0.0788171	newly 5 weeks spec	5505.	5249.31	{3076., 7088.}	25.9599	0.125397	newly 5 weeks cumul	13 758.	13 582.6	{8561., 17 834.}	22.2532	0.103232	newly 59 weeks cumul	89 914.	57 456.2	{34 091., 81 977.}	36.1219	0.229999
27	newly 3 weeks spec	3178.	4053.91	{3715., 4308.}	28.203	0.106568	newly 3 weeks cumul	6240.	7576.05	{6973., 7947.}	21.846	0.0846256	newly 5 weeks spec	6900.	8691.48	{8170., 9078.}	26.8023	0.103024	newly 5 weeks cumul	18 218.	22 667.8	{20 959., 23 767.}	25.0891	0.0966027	newly 60 weeks cumul	90 939.	84 058.1	{53 222., 92 749.}	10.0936	0.0542297
28	newly 3 weeks spec	1642.	3162.69	{2101., 4152.}	94.2101	0.26882	newly 3 weeks cumul	2833.	4846.35	{3367., 6267.}	73.1518	0.222787	newly 5 weeks spec	6075.	11 731.1	{7834., 14 486.}	95.2815	0.282625	newly 5 weeks cumul	12 201.	23 472.5	{16 038., 29 866.}	94.1627	0.276676	newly 64 weeks cumul	93 780.	83 190.6	{56 873., 92 471.}	11.3021	0.0598612
29	newly 3 weeks spec	2186.	2180.78	{1246., 3343.}	28.2845	0.168928	newly 3 weeks cumul	4260.	4391.58	{2674., 6560.}	26.816	0.13977	newly 5 weeks spec	5160.	4019.84	{2522., 5637.}	27.5981	0.193424	newly 5 weeks cumul	12 858.	11 527.9	{7205., 16 788.}	25.6391	0.154038	newly 58 weeks cumul	82 934.	35 984.9	{32 884., 39 761.}	56.6102	0.394438
30	newly 3 weeks spec	404.	846.6	{9., 1144.}	214.079	0.822507	newly 3 weeks cumul	607.	1142.52	{53., 1613.}	181.259	0.563334	newly 5 weeks spec	3670.	2509.89	{0., 8759.}	105.635	1.36616	newly 5 weeks cumul	5636.	5248.92	{65., 14 047.}	122.463	0.933639	newly 67 weeks cumul	93 251.	40 575.3	{65., 76 080.}	56.4881	0.880165
31	newly 3 weeks spec	169.	608.78	{1., 277.}	396.189	1.10683	newly 3 weeks cumul	246.	858.94	{6., 335.}	381.919	0.87644	newly 5 weeks spec	2181.	1631.79	{0., 3388.}	126.591	1.63637	newly 5 weeks cumul	3075.	3288.19	{8., 5540.}	156.05	1.33281	newly 68 weeks cumul	88 759.	66 304.5	{9., 93 163.}	27.1949	0.613943
32	newly 3 weeks spec	874.	3942.39	{2447., 4999.}	351.074	0.623802	newly 3 weeks cumul	1385.	5490.29	{3429., 6878.}	296.411	0.567398	newly 5 weeks spec	4137.	16 560.4	{12 623., 18 929.}	300.3	0.596253	newly 5 weeks cumul	7503.	31 925.5	{23 289., 38 465.}	325.503	0.619694	newly 65 weeks cumul	77 539.	90 644.4	{91 273., 93 861.}	21.0716	0.0862202
33	newly 3 weeks spec	1347.	1672.59	{998., 2411.}	34.1418	0.127839	newly 3 weeks cumul	2306.	2820.16	{1841., 3994.}	31.1336	0.116801	newly 5 weeks spec	4307.	5852.77	{3457., 8042.}	45.4495	0.1643	newly 5 weeks cumul	9125.	11 976.9	{7129., 16 307.}	40.1924	0.146667	newly 63 weeks cumul	92 452.	69 684.2	{52 957., 91 181.}	24.6266	0.137604
34	newly 3 weeks spec	2211.	2345.23	{1635., 2890.}	18.1054	0.0779671	newly 3 weeks cumul	4652.	4893.8	{3428., 5819.}	17.1406	0.0744535	newly 5 weeks spec	4361.	4337.22	{3100., 5829.}	17.4946	0.0777805	newly 5 weeks cumul	12 123.	12 540.7	{8891., 15 720.}	16.5498	0.0723879	newly 58 weeks cumul	74 850.	48 817.6	{33 360., 85 729.}	40.0314	0.232868
35	newly 3 weeks spec	845.	1122.82	{741., 1530.}	38.9112	0.130403	newly 3 weeks cumul	1377.	1776.57	{1236., 2358.}	34.9564	0.120541	newly 5 weeks spec	3852.	5172.6	{3295., 6941.}	40.0395	0.134628	newly 5 weeks cumul	7034.	9510.36	{6052., 12 903.}	40.6326	0.135181	newly 63 weeks cumul	93 735.	85 721.4	{71 572., 89 700.}	8.54917	0.0403678
36	newly 3 weeks spec	2415.	2175.33	{1457., 2904.}	21.6948	0.106323	newly 3 weeks cumul	4659.	4304.91	{2874., 5396.}	19.1451	0.0911172	newly 5 weeks spec	5307.	4461.68	{2866., 5644.}	22.1142	0.115507	newly 5 weeks cumul	13 623.	12 036.7	{8347., 15 452.}	20.2231	0.101281	newly 61 weeks cumul	83 417.	49 045.3	{33 518., 89 943.}	44.1487	0.27795
37	newly 3 weeks spec	569.	427.63	{23., 878.}	83.2601	0.707802	newly 3 weeks cumul	794.	652.29	{62., 1214.}	75.0642	0.4852	newly 5 weeks spec	3995.	2313.03	{0., 6834.}	84.1344	1.0975	newly 5 weeks cumul	6432.	4058.76	{127., 9901.}	83.889	0.790578	newly 66 weeks cumul	88 408.	56 445.4	{127., 93 546.}	39.5469	0.649597
38	newly 3 weeks spec	174.	2261.43	{0., 4966.}	1266.24	1.0743	newly 3 weeks cumul	245.	2943.68	{46., 6532.}	1150.49	0.782519	newly 5 weeks spec	2064.	5402.94	{0., 15 350.}	257.581	1.39043	newly 5 weeks cumul	2893.	12 697.7	{48., 35 490.}	423.566	0.998482	newly 66 weeks cumul	81 546.	33 950.5	{48., 67 060.}	58.5633	0.996222
39	newly 3 weeks spec	1689.	2106.31	{1124., 3006.}	39.5009	0.161527	newly 3 weeks cumul	3181.	3959.33	{2013., 5922.}	39.3879	0.159138	newly 5 weeks spec	4543.	4992.17	{3424., 6363.}	23.9122	0.109452	newly 5 weeks cumul	10 645.	12 382.5	{7649., 15 647.}	30.5513	0.132848	newly 60 weeks cumul	70 294.	56 106.5	{35 309., 92 707.}	34.3053	0.189579
40	newly 3 weeks spec	2733.	2529.36	{1389., 3833.}	29.1599	0.185347	newly 3 weeks cumul	5234.	4942.55	{2778., 7319.}	27.5019	0.158775	newly 5 weeks spec	6145.	4783.09	{3023., 6277.}	25.3082	0.183797	newly 5 weeks cumul	15 669.	13 470.2	{8085., 19 215.}	25.2576	0.162365	newly 61 weeks cumul	84 270.	35 893.7	{33 777., 37 300.}	57.4063	0.405018
41	newly 3 weeks spec	2085.	1848.05	{792., 3073.}	37.1803	0.212446	newly 3 weeks cumul	3973.	3584.06	{1614., 5706.}	34.0956	0.192443	newly 5 weeks spec	5334.	4016.74	{1928., 6559.}	37.2883	0.228922	newly 5 weeks cumul	12 888.	10 490.7	{4867., 17 241.}	35.5826	0.210215	newly 62 weeks cumul	72 211.	37 740.4	{33 055., 52 755.}	47.736	0.286661
42	newly 3 weeks spec	641.	517.42	{42., 827.}	88.624	0.626304	newly 3 weeks cumul	992.	808.31	{126., 1342.}	78.3034	0.472947	newly 5 weeks spec	4341.	2220.55	{47., 4389.}	86.7687	1.00222	newly 5 weeks cumul	7191.	4273.55	{247., 7419.}	86.6818	0.754036	newly 66 weeks cumul	73 182.	43 264.7	{11 455., 86 550.}	46.2077	0.463794
43	newly 3 weeks spec	1358.	2374.57	{1647., 3179.}	77.8387	0.240136	newly 3 weeks cumul	2359.	3801.83	{2639., 4939.}	64.677	0.20889	newly 5 weeks spec	4809.	8805.51	{5836., 10 951.}	84.8228	0.257936	newly 5 weeks cumul	9757.	17 622.	{12 561., 22 824.}	82.6473	0.252189	newly 59 weeks cumul	92 457.	68 846.3	{56 570., 71 238.}	25.537	0.129544
44	newly 3 weeks spec	944.	2824.74	{1468., 4344.}	199.407	0.424365	newly 3 weeks cumul	1464.	3780.58	{2066., 5957.}	158.661	0.365894	newly 5 weeks spec	6185.	15 562.8	{10 744., 21 044.}	151.622	0.387831	newly 5 weeks cumul	10 162.	27 512.9	{17 290., 38 343.}	170.743	0.409374	newly 65 weeks cumul	91 937.	90 147.2	{78 912., 93 881.}	4.13359	0.0194608
45	newly 3 weeks spec	599.	517.	{44., 1027.}	125.078	0.787633	newly 3 weeks cumul	910.	690.24	{97., 1269.}	110.303	0.693358	newly 5 weeks spec	4808.	3056.16	{148., 11 395.}	117.039	1.05733	newly 5 weeks cumul	7429.	5424.97	{318., 17 535.}	122.708	0.948068	newly 64 weeks cumul	82 950.	85 356.3	{78 213., 92 234.}	8.67242	0.0684222
46	newly 3 weeks spec	564.	331.63	{148., 535.}	49.4486	0.32044	newly 3 weeks cumul	898.	532.96	{257., 826.}	47.755	0.303792	newly 5 weeks spec	3017.	1770.59	{722., 2763.}	51.1293	0.331055	newly 5 weeks cumul	5294.	3089.36	{1342., 4792.}	50.6759	0.326512	newly 64 weeks cumul	85 120.	86 423.3	{71 378., 93 820.}	8.11152	0.0373525
47	newly 3 weeks spec	765.	4368.66	{1686., 7751.}	471.067	0.638261	newly 3 weeks cumul	1158.	5734.35	{2274., 9794.}	395.194	0.581598	newly 5 weeks spec	3839.	17 653.4	{12 104., 24 970.}	359.844	0.644662	newly 5 weeks cumul	6738.	34 410.7	{20 597., 54 851.}	410.696	0.675571	newly 66 weeks cumul	82 631.	87 131.1	{67 980., 93 829.}	11.1089	0.0478301
48	newly 3 weeks spec	268.	712.54	{177., 1321.}	179.955	0.419661	newly 3 weeks cumul	370.	971.8	{250., 1738.}	175.281	0.397003	newly 5 weeks spec	2437.	6110.01	{1505., 10 616.}	168.146	0.432762	newly 5 weeks cumul	3631.	9406.88	{2331., 16 443.}	174.999	0.415635	newly 66 weeks cumul	91 693.	86 521.8	{73 588., 93 760.}	7.23777	0.0702387
49	newly 3 weeks spec	151.	5096.15	{0., 13 960.}	3321.93	1.22905	newly 3 weeks cumul	195.	5946.12	{25., 14 727.}	2988.51	0.983483	newly 5 weeks spec	3032.	10 460.9	{0., 28 178.}	313.923	1.1709	newly 5 weeks cumul	3868.	26 664.9	{25., 75 952.}	653.161	1.05054	newly 68 weeks cumul	88 820.	64 750.	{25., 92 193.}	28.7649	0.569153
50	newly 3 weeks spec	2772.	2825.32	{2658., 2980.}	3.97835	0.0170106	newly 3 weeks cumul	5085.	5091.77	{4824., 5364.}	3.43382	0.0149275	newly 5 weeks spec	6948.	7416.53	{7115., 7650.}	6.85161	0.0285987	newly 5 weeks cumul	16 731.	17 355.5	{16 658., 18 089.}	4.24189	0.017922	newly 59 weeks cumul	91 561.	92 847.2	{92 366., 93 203.}	1.40474	0.00605551
